# Tocilizumab in COVID-19: A Double-Edged Sword?

**DOI:** 10.3390/biomedicines12122924

**Published:** 2024-12-23

**Authors:** Bartosz Kudliński, Jacek Zawadzki, Wiktoria Kulińska, Jagoda Kania, Magdalena Murkos, Marta Stolińska, Dominika Zgoła, Anna Noga, Paweł Nowak

**Affiliations:** Department of Anesthesia, Critical Care and Rescue Medicine, Collegium Medicum, University in Zielona Góra, 65-046 Zielona Góra, Poland; jacekzawadzkimed@gmail.com (J.Z.); wikaaa94@gmail.com (W.K.); kaniajag@gmail.com (J.K.); magdalenamurkos@gmail.com (M.M.); mrtstolinska@gmail.com (M.S.); dominika.zgola@gmail.com (D.Z.); annmaria1605@gmail.com (A.N.); nowak.pawel970@gmail.com (P.N.)

**Keywords:** COVID-19, SARS-CoV-2, tocilizumab, cytokines, steroids, NDM, *A. baumanii*

## Abstract

**Background/Objectives:** SARS-CoV-2 was responsible for the global pandemic. Approximately 10–15% of patients with COVID-19 developed respiratory failure with adult acute respiratory distress syndrome (ARDS), which required treatment in the Intensive Care Unit (ICU). The cytokine storm observed in severe COVID-19 was frequently handled with steroids. Synergically, tocilizumab, an anti-interleukin-6 receptor monoclonal antibody, gained popularity as a cytokine storm-suppressing agent. However, immunosuppression was proven to increase the predisposition to infections with resistant bacteria. Our study aimed to assess the relationship between positive tests for secondary infections and the survival of patients with severe COVID-19-attributed ARDS treated with immunosuppressive agents. **Methods:** This study included 342 patients qualified for the ICU and mechanical ventilation (MV). The patients were divided based on the type of immunomodulating therapy and the culture tests results. **Results:** The results showed the highest survival rate among patients <61 years, favoring the combined treatment (tocilizumab + steroids). Atrial fibrillation (AF) and coronary heart disease (CHD) correlated with a lower survival rate than other comorbidities. Tocilizumab was associated with an increased risk of positive pathogen cultures, which could potentially cause secondary infections; however, the survival rate among these patients was higher. **Conclusions:** MV and ICU procedures as well as the application of tocilizumab significantly decreased the mortality rate in patients with severe COVID-19-related ARDS. The suppression of cytokine storms played a crucial role in survival. Tocilizumab was found to be both efficient and safe despite the ‘side effect’ of the increased risk of positive results for secondary infections.

## 1. Introduction

In December 2019, the severe acute respiratory syndrome coronavirus 2 (SARS-CoV-2) pandemic broke out in Wuhan and spread worldwide in a period shorter than nine months. According to the WHO estimates, over 774 million cases of SARS-CoV-2 infection and 7 million COVID-19-related deaths have been confirmed thus far [1]. The broad spectrum of COVID-19 clinical manifestations ranges from an asymptomatic course to acute respiratory distress syndrome (ARDS) and death [2]. The most severe cases of fully developed ARDS require mechanical ventilation (MV) and treatment in the Intensive Care Unit (ICU). Hyperinflammatory COVID-19-related pneumonia is connected with an increased level of inflammatory pleiotropic cytokine interleukin 6 (IL-6) and an outbreak of the cytokine storm [3,4]. Il-6 plays a crucial role in signaling the cytokine storm, by acting via two main pathways: classical signaling, which includes binding to membrane-bound IL-6 receptors (IL-6R) and activation of the Janus kinase (JAK)—STAT3 pathway, and trans-signaling, where IL-6 binds to soluble IL-6R, which facilitates the transmission of the signal in cells lacking membrane-bound receptors. The latter pathway is characteristic for pro-inflammatory responses and takes part in driving the systemic inflammation present in COVID-19. The treatment with anti-inflammatory and immunosuppressive drugs, such as steroids, was commonly provided to limit the fulminant course of the cytokine storm. An increased IL-6 level was described as one of the promising biomarkers predicting mortality in COVID-19-related ARDS [5]. This cytokine indicatory potential was also used to introduce and conduct specific anti-IL-6R pharmacotherapy in the form of a humanized monoclonal antibody in the class of IgG1, i.e., tocilizumab. The mechanism of its acting can be explained by inhibiting the binding of IL-6 to its receptor. As a result, tocilizumab blocks both pathways, thereby reducing IL-6-driven hyperinflammation, which is crucial in managing severe COVID-19 complicated by ARDS. According to the current guidelines (WHO, NIH), tocilizumab has become part of the treatment protocols as an adjunct corticosteroid in reducing hyperinflammation in severe COVID-19. Alas, the main disadvantage of this kind of treatment is that tocilizumab non-selectively blocks both anti-inflammatory and pro-inflammatory actions of IL-6. As a result, the IL-6 protective function may also be inhibited, which is a predisposing factor for secondary bacterial infections (SIs), especially when the treatment is provided in combination with steroid therapy [6]. We hypothesized that the combined therapy with tocilizumab and steroids in adequate doses aimed at suppressing the cytokine storm in severe COVID-19-related ARDS is associated with improved prognosis among patients undergoing intensive care.

### Objective

This study aimed to show that immunosuppressive treatment with tocilizumab was more effective than steroid therapy alone, and positive results for secondary infections did not affect the patient’s prognosis, which indicated the therapy’s safety.

## 2. Materials and Methods

Our study was based on a prospectively analyzed database of 367 adult patients who qualified for intensive care procedures. Twenty-five patients were excluded due to incomplete clinical data, leaving 342 patients for analysis. All were admitted to the Intensive Care Unit (ICU) at the University Hospital in Zielona Góra, Poland, between December 2020 and June 2021, with observation periods ranging from 0 to 54 days. All patients were unvaccinated due to various reasons. The RT-PCR of nasopharyngeal swabs confirmed SARS-CoV-2 infection. ICU admission and inclusion in this study were based on the Berlin criteria, modified by Villae et al., including the need for mechanical ventilation due to ARDS, symptom onset within one week or worsening respiratory symptoms, and HRCT findings of ground-glass opacities and progressive lesions [7,8,9]. Oxygenation status was assessed as PaO_2_/FiO_2_ ratios of 200–300 (mild ARDS), 100–200 (moderate ARDS), and <100 (severe ARDS) with PEEP ≥ 5 cm H_2_O.

Steroid therapy was initiated on Day 0 for all patients meeting ARDS criteria. The starting dose was adjusted according to ARDS severity, with mild cases receiving 6 mg/day of dexamethasone, moderate cases 10 mg/day, and severe cases 20 mg/day. However, doses could be escalated or adjusted dynamically within individual patients, depending on clinical progression, oxygenation status (PaO_2_/FiO_2_), and radiological findings. The therapy was continued for a maximum of 14 days or until significant clinical improvement was observed, whichever came first.

Tocilizumab therapy was reserved for patients with severe COVID-19 pneumonia, defined by PaO_2_/FiO_2_ < 300 and progressive lesions on HRCT. It was administered intravenously at a dose of 8 mg/kg (maximum 800 mg) as a single dose, typically within the first 48 h of ICU admission. No additional doses were given, even in cases of limited initial response. Exclusion criteria for tocilizumab included coexisting infections, PaO_2_/FiO_2_ > 300, non-progressive fibrotic lesions on HRCT, and severe organ dysfunction or hematologic disorders. All patients received standardized peri-COVID care, regardless of therapy type. Blood cultures were obtained every 72 h, and bronchoalveolar lavage (BAL) samples were collected every 48 h to monitor secondary infections. Cultures with bacterial concentrations > 10^5^ CFU/mL or clinical symptoms of infection were treated with appropriate antibiotics. Patients with positive culture results were classified into the study group, and patients with negative results were included in the control group. Baseline characteristics, including age, gender, and key comorbidities, were incorporated into the multivariable Cox proportional hazards model to assess factors influencing survival. Vaccination status was not included as a variable in the model, as all patients in this study were unvaccinated due to the inclusion criteria. Hence, this analysis focused on relevant factors without redundancy. The primary endpoint was survival until ICU discharge. Details of the statistical methods and adjustments for confounding variables are provided in the Statistics Section. This study adhered to the Helsinki Declaration and was approved by the Bioethics Commission at the Regional Medical Chamber in Zielona Góra, Poland (No. 21/157/2021).

### Statistics

The statistical methods used included logistic regression with sub-model selection based on the Akaike information criterion (AIC) to model the probability of patient survival. Kaplan–Meier survival curves and the log-rank test were used to assess differences in survival function according to the selected variables. To adjust for potential confounding factors, such as age, gender, comorbidities, and concurrent medications, multivariable logistic regression analyses and a Cox proportional hazard model were employed, allowing for the evaluation of the association between treatment strategies and survival outcomes while accounting for key baseline characteristics. Pearson’s correlation coefficient, the chi-square test, Fisher’s exact test, and Student’s t-test were used to assess relationships between variables. The distributions of variables are presented using box plots for quantitative variables and bar charts for discrete variables. All statistical analyses were performed with statistical significance set at *p* < 0.05.

## 3. Results

Our analysis included 342 patients with the most severe course of COVID-19 who required hospitalization in the ICU and MV. The base model and AIC model are presented in Table 1.

All patients included in this study were unvaccinated due to inclusion criteria, either by personal choice or due to medical contraindications; hence, the vaccination status was not included as a variable in the model. Inflammatory indicators: PCT—procalcitonin assay, CRP—C reactive protein, NEU—neutrophil, PLT—platelet; Comorbidities: AH—arterial hypertension, AF—atrial fibrillation, CHD—coronary heart disease, HF—heart failure, CKD—chronic kidney disease, COPD—chronic obstructive pulmonary disease, DM2—diabetes mellitus 2; Secondary infections: NDM—New Delhi metallo-β-lactamase, MRSA—Methicillin-resistant *Staphylococcus aureus*, GRE—Glycopeptide-resistant *Enterococcus*, VRE—Vancomycin-resistant *Enterococci*.

**Figure 1:**
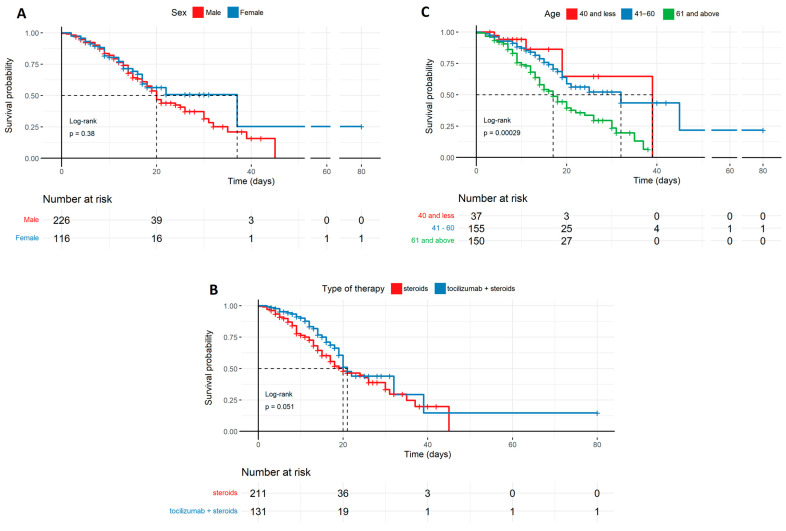
The probability of survival of COVID-19 patients in the ICU in relation to sex, age, and therapy described by the Kaplan–Meyer curves: (**A**) Survival probability by sex, with a trend favoring females, though without sufficient statistical significance. (**B**) Survival probability by therapy, favoring tocilizumab + steroids with marginal statistical significance (*p* = 0.051). (**C**) Survival probability by age, with a trend favoring younger patients, which is statistically significant (*p* < 0.001). Age (years); types of therapy used: steroids, tocilizumab + steroids.

Figure 1 shows three survival probability plots with regard to the following variables: sex, age, and type of treatment. The sex distribution (Figure 1A) did not differ significantly, while the age distribution (Figure 1C) showed a strong correlation with the highest survival rate recorded in patients aged 41–60 years. The type of therapy (Figure 1B) was on the verge of statistical significance (*p* = 0.051), favoring the survival of patients treated with tocilizumab and steroids over patients treated with steroids alone.

**Figure 2:**
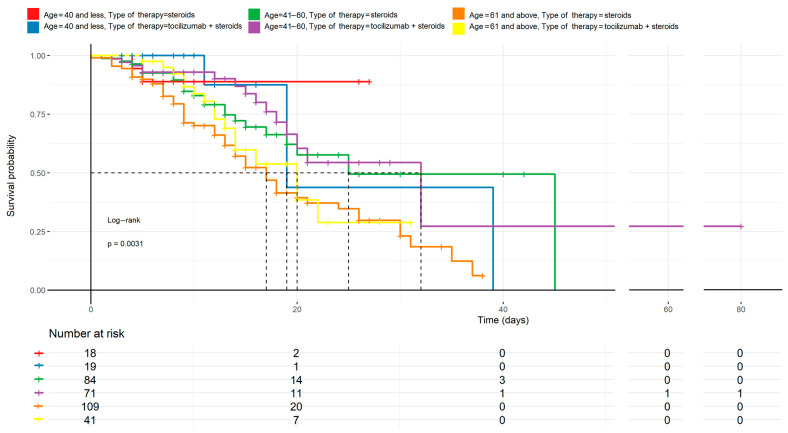
Age comparison to treatment methods described by the Kaplan–Meyer curves. The differences between the curves are statistically significant (*p* < 0.05). Regardless of the therapy used, younger patients have a higher survival chance than older ones. Tocilizumab combined with steroids appears to improve outcomes mostly in middle-aged patients (41–60 years), but the effect is less pronounced in the oldest cohort (≥61 years).

The comparison of the two most statistically significant variables, i.e., age and treatment method, is shown in Figure 2. No statistical differences in survival were observed between the groups of patients ≤40 and >61 years of age according to the treatment method. However, it must be acknowledged that the >61 group was considerably larger than the ≤40 (150 vs. 37 patients, respectively), which shows a significant disproportion by treatment method. The largest and most proportionate group, i.e., patients aged 41–60 years (155 patients), showed a survival advantage in the tocilizumab group compared to the one treated with steroids alone.

**Figure 3:**
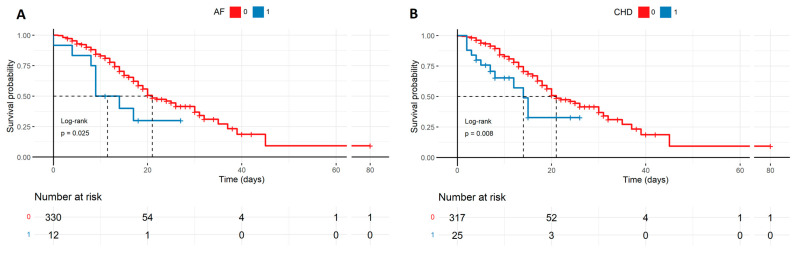
Survival probability of patients with comorbidities such as AF and CHD described by the Kaplan–Meyer curves. The groups of patients suffering from the diseases are smaller in size, though there is a high statistical difference in survival probability favoring patients without CHD (**B**) and AF (**A**) in severe COVID-19 (respectively, *p* = 0.025, *p* = 0.008). AF—atrial fibrillation, CHD—coronary heart disease.

Among the analyzed comorbidities that are presented in Table 1, AF and CHD correlated with a significantly lower survival rate, which is also described by the Kaplan–Meyer curves (Figure 3). Interestingly, patients with diseases of the respiratory system, immune system, cancer, or obesity did not demonstrate a greater statistical value in the survival rate analysis of COVID-19 infection.

**Figure 4:**
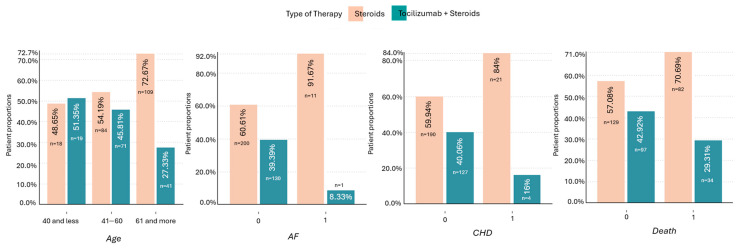
Quality chart of patients showing proportions of age, presence of AF, CHD, and mortality with regard to applied therapy. The therapy including tocilizumab + steroids was more frequently used in patients <40 y.o., without AF and/or CHD. The difference between the therapies (toci + steroids vs. steroids) was greater in patients who died (29.3% vs. 70.7%) compared to patients who survived (42.9% vs. 57.1%). AF—atrial fibrillation, CHD—coronary heart disease.

A qualitative graph of age, AF, CHD, and mortality with regard to the applied therapy is shown in Figure 4.

**Figure 5:**
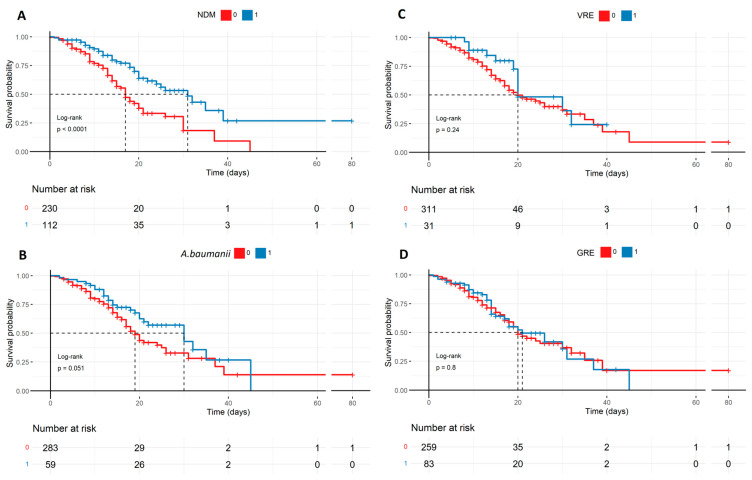
Potential secondary infections in all patients described by the Kaplan–Meyer curves: (**A**) The survival probability regarding NDM infection favoring patients with NDM, which is statistically significant (*p* < 0.0001). The statistical significance is marginal (*p* = 0.051) in the case of *A. baumanii* (**B**) with the same trend, though there is no statistical significance in mortality prediction in the case of VRE and GRE (**C**,**D**). The patients with NDM are also the most proportional compared to the patients without secondary infection regarding numbers, meaning it was the most popular SI. NDM—*Klebsiella pneumoniae* New Delhi metallo-β-lactamase-resistant, VRE—Vancomycin-resistant Enterococcus, *A. baumanii—Acinetobacter baumanii*, and GRE—Glycopeptide-resistant Enterococcus.

**Figure 6:**
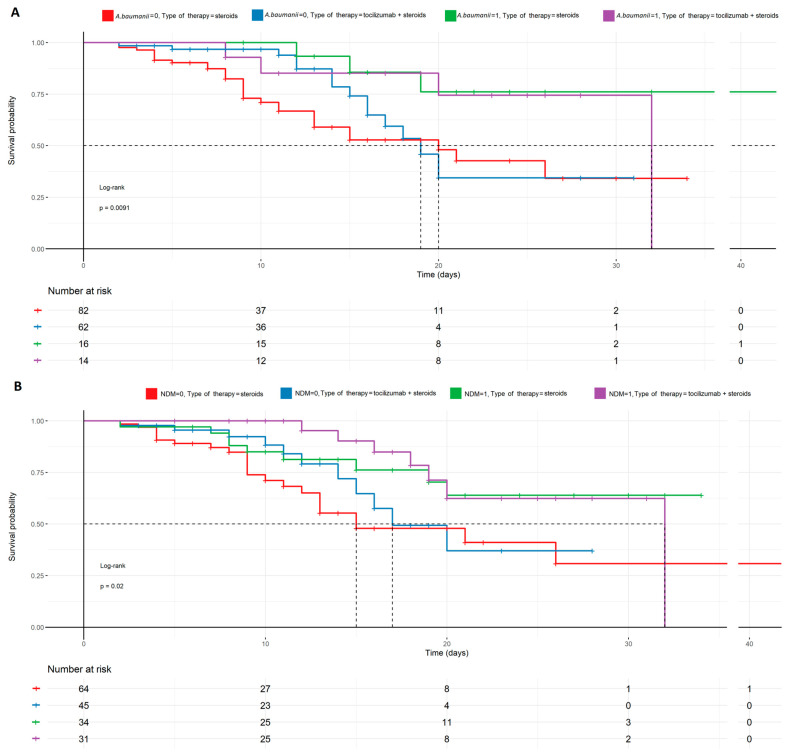
Probability of survival in potential secondary infections with *A. baumanii* and NDM and the therapy used, described by the Kaplan–Meyer curves. There is a statistical significance in the case of mortality prediction in NDM (**B**) and *A. baumanii* (**A**) in regard to the therapy used (*p* < 0.009, *p* < 0.02), favoring patients treated with tocilizumab + steroids and having NDM (+) or *A. baumanii* (+). On the contrary, patients treated with steroids only, with no secondary infections, were more likely to die. NDM—*Klebsiella pneumoniae* New Delhi metallo-β-lactamase-resistant, and *A. baumanii—Acinetobacter baumanii.*

Tocilizumab was observed to increase the risk of secondary infections; however, the mortality in patients with positive test results for such infections was not elevated. Figure 5 shows the graphs of the positive culture of pathogens, potentially causing secondary infections in all patients, while Figure 6 shows statistically significant potential infections, i.e., NDM and *A. baumanii*, dependent on the therapy used.

## 4. Discussion

The key finding of our research is that tocilizumab appeared to be indirectly connected to some potential secondary bacterial infections while also contributing to the decreased mortality rate through its immunomodulatory effects. To understand this multidimensional problem, basic immunologic mechanisms and the phenomenon of cytokine storms in COVID-19 should be accounted for.

### 4.1. Immunologic Mechanisms in COVID-19 and the Cytokine Storm

SARS-CoV-2 is primarily associated with the development of acute respiratory syndrome. However, viral interactions with other organs are also frequent, which is demonstrated by clinically diverse manifestations [10,11]. SARS-CoV-2 was reported to use the superficial cellular receptor ACE2 for entry [12]. However, recent studies identified an alternative pathway via receptor NRP1 [13], which is expressed in various cells and tissues across the body, thereby increasing the virus infectivity [14]. The clinical manifestations are strictly dependent on the host’s immune response, which may vary from mild symptoms to severe and acute conditions such as ARDS and organ failure [15,16]. As in other viral infections, the primal, innate immune response is based on cytokines as pro-inflammatory mediators, but cytokine overproduction leads to a hyper-inflammatory response, which is referred to as a cytokine storm and manifests itself with high severity in COVID-19 [17]. The difference, however, is that, unlike other respiratory viral infections, SARS-CoV-2 induces the release of specific cytokines, which is called a cytokine signature [18]. Patients admitted to the ICU were observed to express elevated factors like the C-X-C motif, CXCL10, IL-2R, C-C motif, CCL2, TNFα, CRP, ferritin, and IL-6, which accelerate and escalate the cytokine storm [19]. The uncontrolled cytokine storm results in T-cell deactivation and delayed B-cell response, a crucial impairment to the immune system functioning [20].

### 4.2. A Perfect Support or a Main Player?

Tocilizumab, an anti-interleukin-6 receptor monoclonal antibody, applied in combination with steroids, could support the therapeutic effect of steroids by inhibiting an IL-6 pathway and decreasing cytokine storm escalation [21]. According to the RECOVERY trial on tocilizumab therapy in patients with severe COVID-19, it significantly reduced the mortality rate compared to the standard care (i.e., corticosteroids, antibiotics, and anticoagulation) [22]. We focused on 562 (14%) of 4116 patients included in the RECOVERY trial who received invasive MV and tocilizumab or standard care (268 vs. 294, respectively). The administration of tocilizumab (8/mg/kg), which supported the effect of steroids (dexamethasone, 6 mg), in the groups with non-invasive ventilation was found to significantly decrease mortality. However, the mortality rate did not differ significantly between tocilizumab and the standard care groups (49% vs. 51%, respectively) in ICU patients who received MV. Contrastingly, our research recorded a higher survival rate (*p* = 0.051) in critically ill patients treated with steroids and tocilizumab vs. steroids only (Figure 1). While the RECOVERY trial investigated a dexamethasone dose of 6 mg, our study analyzed gradually increasing dexamethasone doses reaching up to 20 mg. Therefore, a question arises: whether such high doses of steroid drugs applied along with tocilizumab also had an impact on our results, which differ so considerably from the RECOVER trial findings. In April 2024, in his letter to the editor of the Lancet, Narasaraju et al. asked about the premature stop due to the safety reasons of RECOVERY trial in terms of determining the efficacy of higher doses (20 mg followed by 10 mg) of dexamethasone compared to inhibitors of IL-6 [23]. The authors inquired about the severity of inflammation when administering higher doses of dexamethasone. They also suggested that dexamethasone may have had an undesirable immunosuppressive impact if it had been used in cases other than severe hyperinflammation. Considering RECOVERY, it is justified to assume that this was the case. The beneficial effect of steroids has been confirmed and included in the worldwide standard therapy [24,25]. It has also been argued that higher doses of steroids (>15 mg of dexamethasone) correlated with better outcomes because the glucocorticoid receptors in the cytosol are completely saturated [26]. Nevertheless, the current guidelines for COVID-19 treatment published by the National Institutes of Health (NIH) (updated 10 October 2023) referred to the RECOVERY trial and still recommend the dose of 6 mg of dexamethasone applied for ten days or until discharge from hospital, whichever comes first [26].

### 4.3. Tocilizumab and ICU Admission Criteria

The conflicting results about the survival rates can most likely be attributed to the lack of a unified approach to qualifications for the ICU at that time, e.g., the criteria for tocilizumab administration. According to the current COVID-19 Treatment Guidelines by NIH, tocilizumab or baricitinib are the preferred immunomodulators recommended from the stage when patients have rapidly increasing oxygen needs and systemic inflammation appears (BIIa) [25]. However, the approach was not as unified at the pandemic peak. In 2021, Avni et al. published a meta-analysis of eight randomized studies to evaluate whether tocilizumab decreased mortality in patients with severe COVID-19 infection [21]. It was noticeable that the definitions and scales used to qualify patients for the ICU differed across the analyzed trials. Four studies used the seven-point ordinal score (≥3), one applied the WHO ten-point Clinical Progression Scale (0–10; ≥5), the type of oxygen therapy was used as a scale in one of the studies, and two trials did not use any particular scale for the approach selection. Furthermore, the severity of COVID-19 was described differently: “Severe pneumonia ~37% ventilated at randomization”; “Patients who did not require noninvasive or invasive MV; ~15% hospitalized in ICU at baseline”; “Invasive or non-invasive MV, and/or intravenous infusion of any vasopressor or inotrope”; “Patients receiving supplemental oxygen or MV and had abnormal levels of at least two serum biomarkers (CRP, D dimer, LDH, or ferritin)”.; and “SpO2 < 92% on room air or receiving oxygen therapy, and CRP ≥ 75 mg/L”. It was also indicated that the variability of MV among patients admitted to the ICU ranged from ~30 to 90% across different countries, which makes any conclusions difficult to refer to other relevant studies. Retrospectively, an objective indication of the factor that had a critical impact on the particular patient and whether the qualification timing was optimal are also challenging.

### 4.4. Tocilizumab and Age

Age is one of well-known, critical factors for both qualification for ICU admission and the survival of patients with severe COVID-19 infections. Our comparison of 150 patients aged >61 years and 155 patients aged 41–60 showed that only the former took advantage of the investigated agent (Figure 2). Interestingly, patients younger than 40 years did not benefit from tocilizumab administration either; however, the group was considerably smaller (37 patients), which can definitely be attributed to the fact that younger COVID-19 patients required hospitalization incomparably less frequently than older ones, and, when hospitalized, they manifested milder symptoms. Consistently, Stone et al. reported no significant differences between tocilizumab therapy and the standard care in patients aged >60 years of age [27].

### 4.5. Tocilizumab and Secondary Infections

Tocilizumab is commonly used in rheumatoid arthritis (RA), and secondary infections were also reported in RA patients treated with tocilizumab [28,29]. Tocilizumab-related secondary infections may also be expected in patients with COVID-19. On the other hand, some research included in the meta-analysis by Avni et al. reported that serious infections appeared less frequently after tocilizumab administration, suggesting the contrary tendency. Moreover, the definition of ‘serious infections’ remains vague and under-defined in most studies, which makes compelling evidence harder to obtain. In 2020, Guaraldi et al. investigated the efficacy of tocilizumab therapy in COVID-19-related pneumonia and observed that secondary infections were developed in 24/179 (13%) of patients treated with tocilizumab vs. 4% (14/365) who received standard care [30]. The infections were defined as bloodstream infections, bacterial pneumonia, candidemia, urinary tract infection, Pneumocystis jirovecii pneumonia, invasive aspergillosis, HBV, and HSV-1 reactivation. By contrast, no similar difference was recorded between tocilizumab vs. standard care ICU patients in a cohort study conducted by Gupta et al. (32.3% (140) vs. 31.1% (1085), respectively) [31]. Admittedly, the patients were carefully monitored for secondary infections, but the authors did not specify the kind of infection or colonization that they diagnosed. Based on our clinical observations, we precisely focused on the presence of opportunistic infections/colonization such as *Klebsiella pneumoniae* New Delhi (NDM), Acinetobacter baumanii (*A. baumanii*), Vancomycin-Resistant Enterococcus (VRE), Glycopeptide-resistant Enterococcus (GRE). Surprisingly, the survival rate was higher among patients with *A. baumanii*, especially with NDM, but no statistical difference was detected in patients with VRE and GRE (Figure 5). Figure 6 shows other correlations, which shed more light on that peculiarity. Tocilizumab applied in combination with high doses of steroids resulted in a higher survival rate despite an elevated risk of positive cultures, potentially causing secondary infections. Apparently, the cytokine storm reduction is the most crucial factor for patient survival despite ‘the side effect’ of secondary pathogen colonization, which represents a valuable finding of our study and contributes significantly to the understanding of this field.

### 4.6. Comorbidities

With regard to comorbidities, AF and CHD were the most significant for the survival rates (Figure 3); however, the number of patients with confirmed AF and CHD was relatively small in our study group (12 and 25, respectively). The investigation on tocilizumab therapy by Biran et al. included 630 ICU patients with COVID-19 and reported that arrhythmia (77 patients, *p* = 0.023), renal failure (76, *p* = 0.015), and coronary disease (132, *p* = 0.11) were the most unlikely to be treated with tocilizumab [32]. On the other hand, the authors also indicated that the number of three or more comorbidities per se was highly significant for a no-tocilizumab treatment decision (comorbidity count *p* = 0.025). Consequently, tocilizumab was a less frequent therapy if one of the diseases occurred as well as when it belonged to a group of three conditions, and the diseases, such as arrhythmia (most frequently AF), renal failure, or coronary disease, are known to most often co-occur in elderly patients with poorer prognosis.

### 4.7. Study Limitations

The main limitation of our study is the heterogeneity of the patients included in the analyses. This was a single-center study, with a limited sample size, which may restrict the generalizability of the findings. The retrospectively collected data are potentially connected with inconsistences or gaps in the information (25 patients were excluded due to incomplete data). Furthermore, observational studies can potentially be influenced by confounding factors such as unmeasured variables or selection bias.

## 5. Conclusions

Our findings support the hypothesis that therapy based on tocilizumab and steroids is associated with improved prognosis in patients with severe COVID-19-related ARDS treated in the Intensive Care Unit. However, due to this study’s observational nature, further controlled trials are needed to confirm causality and refine therapeutic recommendations.

1. The use of tocilizumab was observed to decrease the mortality rate in critically ill patients with severe COVID-19-related ARDS who qualified for ICU procedures and MV.
2. Suppression of the cytokine storm appears to be crucial in the survival of patients with severe COVID-19-related ARDS. This interpretation is supported by the well-documented mechanisms of IL-6 blockade with tocilizumab and the observed trends in our study.
3. As an immunomodulating agent, tocilizumab was a safe treatment option in severe COVID-19 infection.
4. The increased risk for positive cultures of pathogens potentially responsible for secondary infections was the side effect of tocilizumab administration.
5. The positive cultures of pathogens potentially responsible for secondary infections were not connected with the ICU patient survival rate.

Take Home Message:

Tocilizumab is a safe immunomodulatory drug that can limit the frequency and severity of the cytokine storm in infections—in this case, SARS-CoV-2. Its effect on modulating and limiting the inflammatory response may increase the risk of secondary infections, but this phenomenon does not affect the mortality of patients in the ICU.

## Figures and Tables

**Table 1:** Base model and AIC model.

	Cox Model			AIC Selection Cox Model		
Predictors	HR	CI	*p*	HR	CI	*p*
Baseline characteristics and therapy						
Sex [Female]	0.8192	0.517–1.2972	0.395			
Age (years) [41–60]	1.5499	0.5613–4.2801	0.398	1.312	0.5088–3.3840	0.574
Age (years) [>60]	2.7426	1.0022–7.5054	0.050	2.601	1.0249–6.5983	0.044
Tocilizumab + steroids	0.9417	0.5995–1.4793	0.794			
Secondary infections and inflammatory parameters						
NDM	0.5965	0.3840–0.9268	0.022	0.599	0.3910–0.9164	0.018
GRE	0.7566	0.4829–1.1854	0.223			
VRE	0.7217	0.3640–1.4310	0.350			
*A. baumanii*	0.8889	0.5368–1.4720	0.647			
PCT	1.0614	1.0107–1.1146	0.017	1.066	1.0193–1.1143	0.005
CRP	1.0009	0.9990–1.0028	0.356			
NEU	1.0587	1.0234–1.0953	0.001	1.061	1.0268–1.0968	<0.001
PLT	0.9974	0.9956–0.9993	0.006	0.997	0.9953–0.9990	0.002
Comorbidities						
Obesity	0.7831	0.5204–1.1784	0.241			
AH	1.0401	0.6783–1.5950	0.857			
AF	1.0219	0.4519–2.3107	0.958			
CHD	1.9252	0.8585–4.3175	0.112	1.778	0.9139–3.4582	0.090
HF	0.5216	0.1386–1.9635	0.336			
CKD	2.1045	0.8030–5.5153	0.130	2.141	0.9034–5.0724	0.084
COPD	1.3960	0.5099–3.8220	0.516			
Asthma	1.7959	0.7661–4.2100	0.178			
DM2	1.3534	0.7931–2.3096	0.267			
Thyroid diseases	0.3729	0.1424–0.9766	0.045	0.439	0.1830–1.0540	0.065
Immunosuppression	2.7168	0.3387–21.791	0.347			
Cancer	0.7979	0.3021–2.1069	0.649			
Autoimmune disease	1.1245	0.4553–2.7775	0.799			
Observations	342			342		
R^2^ Nagelkerke	0.205			0.182		
Vaccination status	Unvaccinated 100%					

## Data Availability

The data that support the findings of this study are available upon request from the corresponding author [Wiktoria Kulińska].
